# Bacterial quorum sensing compounds are important modulators of microbe-plant interactions

**DOI:** 10.3389/fpls.2014.00131

**Published:** 2014-04-08

**Authors:** Anton Hartmann, Michael Rothballer, Burkhard A. Hense, Peter Schröder

**Affiliations:** ^1^Research Unit Microbe-Plant Interactions, Department of Environmental Sciences, Helmholtz Zentrum München, German Research Center for Environmental Health (GmbH)Neuherberg, Germany; ^2^Institute of Computational Biology, Helmholtz Zentrum München, German Research Center for Environmental Health (GmbH)Neuherberg, Germany

**Keywords:** Quorum sensing, N-acyl-homoserine lactones, systemic responses, innate immunity, mutualists, endophytic bacteria

## Plant—microbiome interactions in the light of the holobiontic concept

Higher organisms evolved in the omnipresence of microbes, which could be of pathogenic or symbiotic nature. A framework of response patterns evolved which is known as innate immunity. A major part of this response is the recognition of microbial-associated molecular patterns (MAMP) such as chitin or lipochitooligosaccharides, peptidoglycan, lipopolysaccharides or flagellum structures, and the initiation of efficient plant defence reactions (Janeway and Medzhitov, 2002; Jones and Dangl, 2006). However, there are many plant-associated endophytic bacteria known, which are living within plants without triggering persistent and apparent defence responses or visibly do not harm the plant. In some cases, even a stimulation of plant growth due to the presence of specific players within the plant microbiome was reported (Turner et al., 2013). It is now generally accepted, that plant performance and activities can only be characterized and understood completely, if the “holobiont,” the plant plus the intimately associated microbiota, is considered (Zilber-Rosenberg and Rosenberg, 2008). The evolutionary advantage of an integrated holobiontic system is characterized by a much better adaptability and flexibility towards rapidly changing adverse environmental conditions. It is still mostly unknown, which particular plant genetic loci are controlling the interactions with the plant microbiome and which signals are steering this cooperativity. Mutualistic microbes are able to overcome or short-circuit plant defence responses to enable successful colonization of the host (Zamioudis and Pieterse, 2012; Alqueres et al., 2013). Beneficial associations with microbes other than mycorhiza or *Rhizobia* are also controlled by systemically regulated or autoregulated processes on top of the basic innate immunity response. The induction of systemic immunity responses like ISR (induced systemic resistance) by some beneficial rhizosphere bacteria or the SAR (systemic acquired resistance) response provoked by pathogens are results of multiple response cascades employed by the plant host to respond to microbial and other environmental interactions. However, the entire response network is by far not yet revealed. For example, bacteria-induced plant responses resulting in improved resistance towards pathogens can also be due to the perception of secondary metabolites, like the surfactin lipopeptide, produced by certain biocontrol Bacilli (Garcia-Gutiérrez et al., 2013) or volatile compounds of plant-associated microbes (Yi et al., 2010). The biocontrol activity of microbial inoculants is probably due to multiple effects of their secondary metabolites to achieve direct inhibition of the pathogenic counterpart as well as an increase of systemic resistance of the plant host.

## Bacterial quorum sensing molecules like *N*-acyl homoserine lactones modulate plant responses toward contact with bacteria

It is hypothesized, that eukaryotic organisms developed ways to sense microbes in addition to the recognition of their MAMPs by their diffusible small molecules. A very ancient and basic feature of unicellular bacteria is their way of environmental sensing and social communication. In many Gram-negative bacteria the synthesis of autoinducers of the *N*-acyl-homoserine lactone (AHL) type is tightly regulated in response to cell density or the cell “quorum” (Eberl, 1999). These metabolites are released into the cellular environment to sense the quality of the ecological niche in terms of diffusion space and the density and distribution of their own population. This environmental sensing mechanism helps to adapt the regulation of their gene expression to the given conditions in their habitat and thus optimizes the fitness of the population. Therefore, the generally known term “quorum sensing” (QS) was supplemented by the more broadly defined concept of “efficiency sensing” (Hense et al., 2007). Since this optimization of *in situ* gene expression is of very basic importance, autoinducer QS-molecules are widespread among bacteria and have quite different molecular structures. AHL are common in Gram-negative bacteria, while cyclic peptides as QS-signals are only to be found in Gram-positive bacteria. The detailed structure of the AHL-molecules can vary; the acyl side chain consists of 4–14 carbon atoms and may also contain double bonds. The C3-atom can be hydroxylated or oxidized to a carbonyl-carbon; thus, considerable information and quite different physicochemical properties can be present within these different AHL-structures. As is outlined below, also plants have obviously learned during their evolution to respond to these QS compounds in different specific ways. We speculate, that QS-compounds are early signals indicating that pathogens are in the surroundings to gather themselves for attack or that mutualists are about to interact with roots.

The first demonstration of specific responses of a plant to bacterial AHLs was demonstrated for the legumes *Phaseolus vulgaris* (Joseph and Phillips, 2003) and *Medicago truncatula* (Mathesius et al., 2003) (Table 1). AHLs from symbiotic (*Sinorhizobium meliloti*) or pathogenic (*Pseudomonas aeruginosa*) bacteria provoked at concentrations as low as nano- to micromolar significant changes in the accumulation of over 150 proteins. Auxin-responsive and flavonoid synthesis proteins were induced and also a secretion of plant metabolites that mimic QS compounds were found, which may have the potential to disrupt QS signaling by associated bacteria. In tomato plants, a specific induction of systemic resistance proteins after inoculation of the roots with C4- and C6-side chain AHL-producing *Serratia liquefaciens* MG1 was discovered independently (Hartmann et al., 2004; Schuhegger et al., 2006). The fungal leaf pathogen *Alternaria alternata* was much less effective, when *S. liquefaciens* MG 1 wild type had been inoculated to roots of tomato plants as compared to the AHL-negative mutant. It could be shown, that salicylic acid was increased as well as SA- and ethylene-dependent defence genes (i.e., PR1a) in MG1-inoculated plants. Furthermore, *Serratia plymuthica* HRO-C48, producing C4-/C6- and OHC4-/OHC6-homoserine lactones, is able to induce ISR-like systemic protection of bean and tomato plants against the fungal leaf pathogen *Botrytis cinnera*; this response was greatly reduced with mutants impaired in AHL-production (Liu et al., 2007; Pang et al., 2009). In contrast, *Arabidopsis thaliana* responds to short (C4- and C6-) *N*-acyl AHL-compounds in a different manner: C4- and C6- homoserine lactones alter the expression of selected hormonal regulated genes which results in changes of the plant's hormone content, in particular an increased auxin/cytokinin ratio (von Rad et al., 2008). However, no systemic resistance response was found to be induced in *A. thaliana* when roots were stimulated with short side-chain AHLs. Ortíz-Castro et al. (2008) found that C10-homoserine lactone elicited developmental changes in the root system in *Arabidopsis* plants by altering the expression of cell division and differentiation-related genes. Furthermore, Liu et al. (2012) and Jin et al. (2012) demonstrated that the root stimulatory effect of C6- and C8- homoserine lactones in *Arabidopsis* plants is mediated through the G-protein coupled receptor encoded by *AtGPA1*. In mung bean, oxoC10-homoserine lactone activates auxin-induced adventitious root formation via H_2_O_2_- and NO-dependent cyclic GMP signaling (Bai et al., 2012). On the other hand, *N*-acyl-AHLs with C12- and C14- side chains induce systemic resistance to the obligate biotrophic fungus *Golovinomyces orontii* in *A. thaliana* and to *Blumeria graminis* f. sp. *hordei* in barley (*Hordeum vulgare*) (Schikora et al., 2011). This response is mediated through altered activation of *AtMPK6*. The mitogen-activated protein kinases AtMPK3 and AtMPK6 were stronger activated by the model elicitor flg22 in the presence of C12- or C14-AHL compounds which resulted in a higher expression of the defence-related transcription factors WRKY26 and WRKY29 as well as the PR1 gene (Schikora et al., 2011). Thus, AHLs with short and medium side chain lengths induce developmental effects on root architecture, while long side chain AHLs induce systemic resistance in *A. thaliana* (Schenk et al., 2012). Furthermore, it was shown, that better water soluble short side chain AHL-compounds are actively taken up into plant roots and transported along the roots into the shoot; in contrast, the lipophilic long acyl side chain AHLs are not transported in barley and *A. thaliana*. (Götz et al., 2007; von Rad et al., 2008; Sieper et al., 2014). However, no uptake was detected in the legume yam bean (*Pachyrhizus erosus* (L.) Urban) (Götz et al., 2007). The latter finding corroborates the report of Delalande et al. (2005) that legumes like *Lotus corniculatus* produce lactonases which degrade AHLs and prevent their uptake and transport. In barley, it could further be demonstrated that C8- and C10-AHLs are taken up in a cell energy dependent manner by ABC-transporters into the root and transported via the central cylinder into the shoot (Sieper et al., 2014).

**Table 1:** **Recent findings of direct AHL impact on different plants**.

**AHL type**	**Plant reaction**	**Plant species**	**References**
Short chain length	Increased transpiration, stomatal conductance	*Phaseolus vulgaris*	Joseph and Phillips, 2003
C6	Primary root elongation	*A. thaliana*	von Rad et al., 2008
C6	Upregulation of metabolism, transport and transcriptional regulation	*A. thaliana*	von Rad et al., 2008
C6 (*Serratia liquefaciens*)	Upregulation of defense genes	*Lycopersicon esculentum*	Schuhegger et al., 2006
C6, C8, C10	Lactonase induction	*Pachyrhizus erosus*	Götz et al., 2007
Oxo-C6, oxo-C8	G-protein coupled receptors for root growth	*A. thaliana*	Jin et al., 2012; Liu et al., 2012
3-oxo-C6 (*Serratia plymuthica*)	Triggering plant immunity	*Cucumis sativa*	Pang et al., 2009
		*Lycopersicon esculentum*	
C6, C8, C10	Root and shoot growth	*Hordeum vulgare*	Götz et al., 2007
3-O-C10	Adventitious root formation	*Vigna radiata*	Bai et al., 2012
C10	Lateral root formation	*A. thaliana*	Bai et al., 2012
C12	Root hair development	*A. thaliana*	Ortíz-Castro et al., 2008
3-oxo-C12 from *P. aeruginosa*	Defense and stress management genes, phytohormones, and metabolic regulation	*Medicago truncatula*	Mathesius et al., 2003
oxo-C12	Resistance induction	*A. thaliana*	Schikora et al., 2011
oxo-C14	Systemic resistance against *Golovinomyces orontii*	*A. thaliana*	Schikora et al., 2011
oxo-C14	Systemic resistance against *Blumeria graminis*	*Hordeum vulgare*	Schikora et al., 2011
3-oxo-C16 from (*Sinorhizobium meliloti*)	Defense and stress management genes, phytohormones and metabolic regulation	*Medicago truncatula*	Mathesius et al., 2003

Interestingly, several plants have been demonstrated to produce AHL-mimic substances or to develop other activities influencing QS of plant associated bacteria (Gao et al., 2003; Bauer and Mathesius, 2004). Flavonoids released by legumes increase the expression of AHL synthesis genes in *Rhizobia* (Pérez-Montano et al., 2011). Indole acetic acid and cytokinin biosynthesis of *Gypsophila* was shown to influence QS, type III secretion system and gall formation activity by *Pantoea plantarum* (Chalupowicz et al., 2009). On the other hand, tobacco plants have been engineered to produce short- and long-side chain AHL-compounds which could be detected in substantial amounts at leaf and root surfaces as well as in soil (Scott et al., 2006). Constitutive expression of QS genes in transgenic tobacco plants leads to alteration in induced systemic resistance elicited by the rhizobacterium *Serratia marcescens* 90–166 (Ryu et al., 2013). Furthermore, transgenic tomato plants engineered to produce different AHL-compounds were demonstrated to alter the activity of plant growth promoting rhizobacteria and resulted, e.g., in increased salt tolerance (Barriuso et al., 2008). We hypothesize, that QS in a plant-microbe holobiont system should be regarded in a bidirectional way with influences from the plant and the microbial partners.

Uptake of AHL-compounds and specific perception of AHLs in animal cells were also studied intensively in recent years (Teplitski et al., 2011; Hartmann and Schikora, 2012). 3-oxo-C12-homoserine lactone (C12-AHL), the major AHL-compound of *Pseudomonas aeruginosa*, was shown to selectively impair the regulation of the nuclear transcription factor NF-κ B which controls innate immune responses in mammalian cells (Kravchenko et al., 2008). C12-AHL also impaired human dendritic cell functions required for priming of T-cells (Bernatowicz et al., submitted). Since the response to AHL-compounds in mammalian systems is complicated due to the interferences with the adaptive immune system, plants provide an ideal model for the detailed interaction studies of basic innate immune responses and developmental processes with *N*-acylhomoserine lactones as modifying bacterial effector molecules.

### Conflict of interest statement

The authors declare that the research was conducted in the absence of any commercial or financial relationships that could be construed as a potential conflict of interest.
